# Arc protein, a remnant of ancient retrovirus, forms virus-like particles, which are abundantly generated by neurons during epileptic seizures, and affects epileptic susceptibility in rodent models

**DOI:** 10.3389/fneur.2023.1201104

**Published:** 2023-07-07

**Authors:** Dmitry A. Sibarov, Vassiliy Tsytsarev, Anna Volnova, Anastasia N. Vaganova, Janaina Alves, Legier Rojas, Priscila Sanabria, Alla Ignashchenkova, Elton D. Savage, Mikhail Inyushin

**Affiliations:** ^1^Sechenov Institute of Evolutionary Physiology and Biochemistry of the Russian Academy of Sciences, Saint Petersburg, Russia; ^2^Department of Anatomy and Neurobiology, University of Maryland School of Medicine, Baltimore, MD, United States; ^3^Institute of Translational Biomedicine, Saint Petersburg State University, Saint Petersburg, Russia; ^4^School of Medicine, Universidad Central del Caribe, Bayamón, PR, United States; ^5^Nevsky Center of Scientific Collaboration, Saint Petersburg, Russia; ^6^Böblingen Dental, Böblingen, Germany

**Keywords:** Arc/Arg3.1, epilepsy, learning, memory, retrovirus, seizures, capsid

## Abstract

A product of the immediate early gene Arc (Activity-regulated cytoskeleton-associated protein or Arc protein) of retroviral ancestry resides in the genome of all tetrapods for millions of years and is expressed endogenously in neurons. It is a well-known protein, very important for synaptic plasticity and memory consolidation. Activity-dependent Arc expression concentrated in glutamatergic synapses affects the long-time synaptic strength of those excitatory synapses. Because it modulates excitatory-inhibitory balance in a neuronal network, the Arc gene itself was found to be related to the pathogenesis of epilepsy. General Arc knockout rodent models develop a susceptibility to epileptic seizures. Because of activity dependence, synaptic Arc protein synthesis also is affected by seizures. Interestingly, it was found that Arc protein in synapses of active neurons self-assemble in capsids of retrovirus-like particles, which can transfer genetic information between neurons, at least across neuronal synaptic boutons. Released Arc particles can be accumulated in astrocytes after seizures. It is still not known how capsid assembling and transmission timescale is affected by seizures. This scientific field is relatively novel and is experiencing swift transformation as it grapples with difficult concepts in light of evolving experimental findings. We summarize the emergent literature on the subject and also discuss the specific rodent models for studying Arc effects in epilepsy. We summarized both to clarify the possible role of Arc-related pseudo-viral particles in epileptic disorders, which may be helpful to researchers interested in this growing area of investigation.

## 1. Introduction

It is known that a significant part (about 8%) of the cell genome in mammals is represented by endogenous retroviruses (ERVs), thus inheriting the ancient germ-line cell infections by retroviruses and the transmission of their genome to the descendants (1–3). While most ERVs are not replication-competent due to broken or tightly controlled viral genes, weakened control can activate ancient retroviral genes and their coded proteins in cancers and senescent cells (4, 5). Besides that, some also retain activity and are important biochemical players in everyday life. For example, ancient viral envelope proteins in the outer cellular layer of the placenta help fusion of the trophoblast cells and became a crucial element in allowing normal pregnancy (6, 7) others determine the fusion of muscle cells and muscle sexual dimorphism (8, 9). Unfortunately, many ERV-related proteins are also implicated in different neurological diseases (10, 11). However, there is not much information regarding their possible role in the pathogenesis of epilepsy.

Actively regulated cytoskeletal-associated (ARC) protein, a protein encoded by the ARC- gene, was characterized in the end of the last century (12, 13). ARC–protein is localized at the synaptic contacts of neurons, and its synthesis depends on the NMDA receptor. This protein plays a critical role in the molecular processes associated with learning and memory and may also serve as a marker of plastic changes in the brain (14, 15).

It is known that the epileptic activity of neurons depends on excitatory-inhibitory synaptic balance (16, 17). Some proteins are well-known regulators of excitatory gain: Pyk2 and Src kinase proteins are the part of NMDA receptor complex (18). PYk2/Src are direct effectors, triggered in active neurons, directly modulating hippocampal excitatory synapses (19). On the other hand, it was determined also that synaptic modulation depends on Arc protein (Activity-regulated cytoskeleton-associated protein, product of endogenous retroviral Arc gene) with recognized activity-dependent expression in neuronal synapses and its expression level is regulated by N-methyl-D-aspartate (NMDA) receptor activation (12). Recently, it was found that Arc protein forms full retrovirus-like capsid in synapses, and may transfer short RNAs from one cell to a post synaptic cell in this way later affecting synaptic morphology (20–22). Interestingly, Arc protein expression changes drastically during epileptic seizures (21, 23).

The study of Arc protein and its synaptic effects is a novel rapidly changing field, but this knowledge is of utmost importance to the understanding of epilepsy because it determines the balance of excitatory and inhibitory synaptic inputs to seizure-prone neurons and the stability of a neuronal network at whole. Here we summarize the emergent literature on this last subject, representing in bullet points Arc's known effects and properties, and then briefly reviewing Arc involvement in epilepsy and relevant experiments in rodent models.

### 1.1. Arc known properties

Mammalian Activity-Regulated Cytoskeleton-Associated protein (Arc, and its variants, products of immediate early Arc gene) is known for its exquisite importance for synapse maturation, synaptic plasticity, learning, and memory, while the dysregulation of Arc expression can have vast consequences for normal brain function, triggering aberrant wiring of neuronal circuits (24, 25). Arc/Arg3.1 mediates activity-dependent elimination of redundant climbing fiber to Purkinje cell synapses in the developing cerebellum (26). In glutamate neurons, following activation of the NMDA receptor, Arc mRNA became significantly upregulated in the nucleus before being transported to the dendrites for translation (12, 27). At postsynaptic sites Arc mRNA localizes in the PSD-95-NMDAR complexes and binds to inactive CaMKIIβ (not bound to calmodulin) (28, 29). Activation of calcium entry via ionotropic glutamate receptors, especially the NMDA subtype, during normal synaptic transmission and in seizures results in calmodulin-dependent dissociation of Arc mRNA from CaMKIIβ and PKA-dependent induction of Arc protein expression (29, 30). Later it happens that spines on dendrites, where Arc was produced, change their morphology increasing the spine density and proportion of thin spines, together with the endocytosis of AMPA receptors leading to decreased synaptic efficacy (21). Arc hyperexpression facilitates not only AMPA endocytosis but also downregulates transcription of the GluA1 subunit of the AMPA receptor which favors synaptic downscaling (31, 32). In this way, Arc participates in synaptic long-term potentiation and the consolidation of long-term memory (33). Interestingly, Arc transcription exhibited distinct temporal kinetics depending on the activation of excitatory inputs that convey functionally distinct information (34).

### 1.2. The mechanism of Arc protein function

Upon hyperexpression, in local dendritic compartments, Arc protein assembles into retrovirus-like capsids packing predominantly Arc mRNA. These capsids leave neurons wrapped in extracellular vesicles and can transmit mRNA to nearby cells (20, 22). Curiously, activation of metabotropic glutamate receptors (mGluR) facilitates Arc mRNA translation in capsid “infected” neurons (20, 22). Arc capsid-mediated transfection was not yet observed in the mammalian brain, but in mice, Arc expression in DRG neurons results in capsid formation modulating skin vasodilation (35). Arc mRNA transfer between cells can probably explain why after hyperactivity Arc accumulates not only in neurons but also in astrocytes, while it is originated from nearby neurons (27, 36, 37). Mammalian Arc protein lacks zinc fingers but has positively charged motifs binding polyanionic mRNA (38–40). This probably allows nonspecific packing of neuronal host mRNAs other than Arc mRNAs, because half of the RNAs encapsidated by retroviruses are host-derived RNAs (41). Extracellular vesicles are supposed to participate in the spread of different neurodegenerative pathologies over time in an activity-dependent manner via synaptic connections (42, 43). Generally, it looks probable that Arc capsids may mediate activity-dependent intercellular paracrine transfer of genetic information, which may alter neighboring cell response to network activity.

### 1.3. Arc protein forms virus-like particles and their history

**Figure 1:**
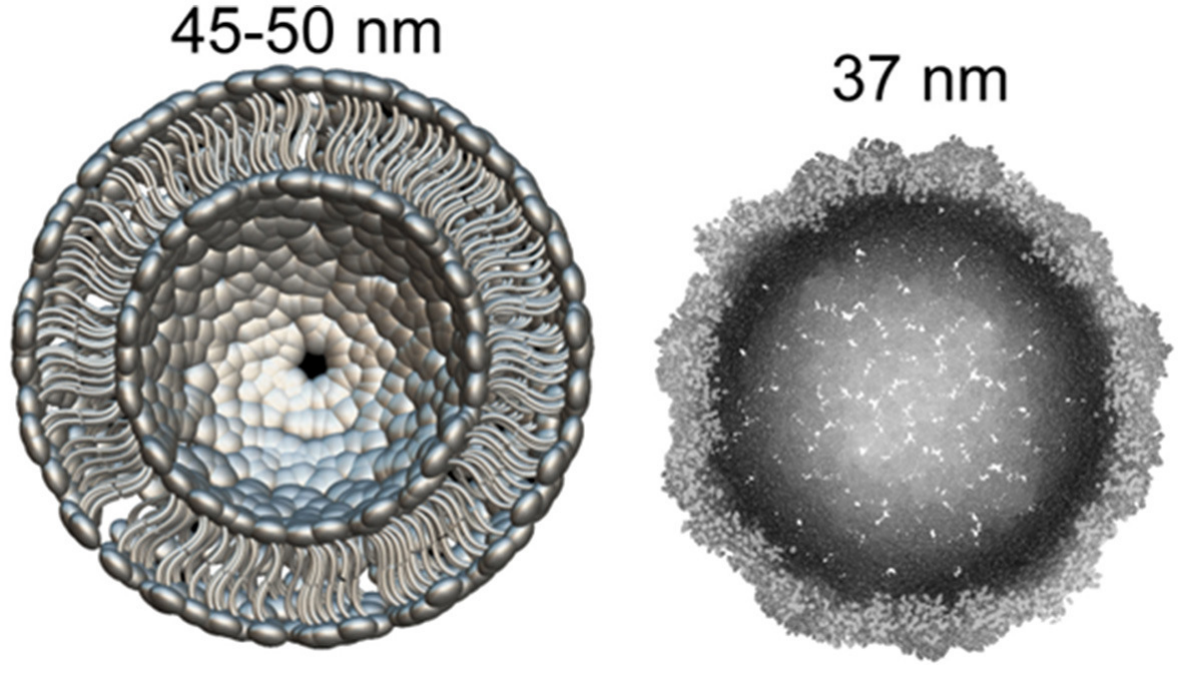
What can be found in synapse: the diameter of glutamatergic synaptic vesicle **(Left)** is approximately 45–50 nM [model drawn according to cryo-EM data by Du et al. (47)]. dArc1 capsid **(Right)**—a viral-like particle made by polymerization of 240 Arc-protein formed capsomeres (47, 48).

Recently it became clear that Arc protein is not only some important regulatory synaptic protein but turns out to be repurposed retrotransposon protein that mediates intercellular mRNA transfer (22, 44, 45). This mechanism involves formation (mainly by glutamatergic neurons) and expression in their synapses of pseudo-viral, retrovirus-like particles (of about 60 nm diameter) made by Arc protein multimers, which encapsulates specific neuronal mRNA and then are trafficked across synaptic boutons (20, 46). The median part of the Arc protein sequence has similarity to modern retroviruses (for example HIV) Group-specific antigen (Gag). Like HIV Gag, Arc forms capsomeres (Figure 1) which self-assemble into capsids of about 30 nM in diameter, while the multimerization of Arc is mediated by its N-terminal helical coil motif (48–50). Arc protein ensembles form multiple capsomeres with symmetric pentameric structures (Figure 1), resembling some viral ion channels (48). Arc variants are found in both birds and humans, but not fish. It is hypothesized that Arc was inserted into the ancestral genome of all tetrapods (amphibians, reptiles, birds, mammals) around 350–400 million years ago. In humans, Arc is found in the greatest amount in brain structures associated with memory, which may be associated with synaptic plasticity and consolidation of memories (44, 51).

Of course, virus-like transport of mRNA can be employed not only in long-term memory, and Arc can be presented better as a multifunctional signaling hub (52). For example, it was shown that Arc can transport mRNAs from mutated genes related to schizophrenia (53). Arc may also play a role in the immunity and activity-dependent β-amyloid generation (30, 54).

### 1.4. Evolution of Arc proteins

Evolutionary analysis shows that Arc is derived from a lineage of Ty3/gypsy retrotransposons, which are also ancestors to retroviruses, that have been repurposed during the evolution to mediate intercellular communication in the nervous system (20, 22). Thus, animals before tetrapods most probably use Ty3/gypsy variants for memory consolidation. On the other hand, the tetrapod Arc protein structure is similar to the one which has been found in Schizophora (true) flies (Dipteros), thus it may have been transferred to a common ancestor of these insects independently (22, 48). At least two Arc homologs (dArc1 and dArc2) are found in flies, which arose by genomic duplication of an ancestral dArc gene but were not detected in any other dipteran (e.g., mosquitoes) or protostome species (20, 22). In Drosophila Arc1 protein forms capsid-like structures that bind mRNA in neurons and are loaded into extracellular vesicles. These vesicles pass from motor neurons to muscles. The disruption of the transfer, in turn, blocks synaptic plasticity (20). Thus, the transsynaptic mechanism of mRNA transport involving retrovirus-like Arc- capsids and extracellular vesicles can be considered proven (55).

While Arc genes originated independently, they still share significant homology in the retroviral Gag domain, and thus the ability to form capsids. Interestingly, the protein participating in memory consolidation in fish retains some immunoreactivity to Arc (56). This domestication of proteins using transposable elements is a well-known phenomenon known as lateral gene transfer that can enrich the recipient and provide a mechanism for evolutionary flexibility (57, 58). About one-half of the mammalian genome consists of DNA with viral or transposon origin and about 8% belong to ancient retroviruses (2, 59). According to the Gene Expression Omnibus (GEO) database for gene expression profiling, the Arc gene was identified as a candidate gene involved in the pathogenesis of various neurological diseases, including epilepsy and a number of others, such as depression.

## 2. Possible involvement of Arc in the mechanisms of neuropsychic disorders

### 2.1. Human brain pathologies associated with ARC-protein

Actively regulated cytoskeletal-associated (ARC) protein, encoded by the ARC gene, was characterized at the end of the last century (12, 13). This protein is localized in the synaptic contacts of neurons, and its synthesis is dependent on the NMDA receptor's activity. This protein plays a critical role in the molecular processes associated with learning and memory and may also serve as a marker of plastic changes in the brain (15).

Impaired Arc protein synthesis is associated with various brain pathologies, including memory disorders, Alzheimer's disease, autism spectrum disorders, schizophrenia, and epilepsy (60–62). Arc cellular pathways have emerged as key regulators of synaptic plasticity, and are becoming known for being central players in genetic risk for many neural disorders (20).

Thus, in a study on a large sample of patients, it was shown that both rare mutations and epigenetic regulation of ARC contribute to the pathogenesis of schizophrenia, at least in some patients (20, 63). It has also been shown that small mutations affecting one or more nucleotides are extremely widespread among glutamatergic postsynaptic proteins, including proteins associated with ARC and NMDAR, regulated by synaptic activity. At the same time, genes affected by mutations in schizophrenia overlap with genes affected by mutations in autism spectrum disorders and some other brain pathologies (64).

Patients with epilepsy have an increased likelihood of experiencing psychotic symptoms, many of which are similar to those of depression. However, many psychiatric comorbidities, not just the symptoms of depression, are more common in patients with epilepsy (65). It remains an open question whether depression is a risk factor for the development of epilepsy.

Interesting results were obtained by measuring the level of Arc/Arg3.1 in the blood plasma of children with a diagnosed autism spectrum disorder (ASDs) (66). The average level of Arc/Arg3.1 protein in blood plasma in autism was significantly higher than in the control (healthy) group. However, no significant association was found between plasma Arc/Arg3.1 protein levels and measures of autism severity (66). This suggests that Arc/Arg3.1 can be used as an early biomarker for diagnosing autism (66).

Autism and Angelman syndrome (AS) share many common characteristics, although Angelman syndrome is not usually included in ASD (67, 68). Angelman Syndrome is a neurological disorder caused by a mutation of the E3 ubiquitin ligase UBE3A, a gene whose mutation is associated with autism spectrum disorders. In childhood, seizures are observed in approximately 80–90% of patients with AS (69). Arc is one of the target proteins of the UBE3A gene. Since Arc is involved in learning and memory, its expression directly affects the manifestation of AS, including the epileptic seizures, typical of this syndrome. But the function of UBE3A during nervous system development and how UBE3A mutations give rise to cognitive impairment in individuals with AS and ASDs remains unclear. Nevertheless, we know that experience-driven neuronal activity induces UBE3A transcription. UBE3A then regulates excitatory synapse development by controlling the degradation of Arc (67). Disruption of UBE3A function in neurons leads to an increase in Arc expression and a concomitant decrease in the number of AMPA receptors at excitatory synapses. This deregulation of AMPA receptor expression at synapses may contribute to the brain pathologies that occur in AS and possibly other ASDs (67). Most researchers agree that the genesis of epileptic seizures in AS has a complex genesis (17). It is known that in patients with AS, there is a global decrease in the volume of a number of subcortical structures and an increase in the volume of gray matter. The degree of the abnormality correlates with the severity of seizures, suggesting that the occurrence of seizures may be directly related to morphological changes in the brain (17).

Fragile X Syndrome (FXS) is one of the most common inherited forms of developmental delay (70). Epilepsy is reported in up to 20% of individuals with fragile X syndrome (71). It was shown that the FMR1 gene, which produces the FMRP protein is responsible for FXS. One of the biological manifestations of FXS is elevated levels of metabotropic glutamate receptor (mGluR)-dependent long-term depression (LTD), (mGluR-LTD) a type of synaptic plasticity which is characterized by a reduction in the synaptic response at the excitatory synapses and overexpression of several proteins including Arc (72, 73). This abnormal overexpression of Arc leads to increased endocytosis of AMPARs and increased mGluR-LTD. In addition, altered dendritic spine morphology was observed not only in animal models of FXS but also in humans with this disorder (70, 74).

Epilepsy does not have a clearly identifiable cause in about half of patients. Also, this disease can be associated with various factors, including various genetic abnormalities. Some types of epilepsy are linked to certain genes, but generally, genetic factors can make a person more sensitive to the mechanisms that cause seizures. In addition, epilepsy has a wide comorbidity with other brain pathologies. Thus, we most likely cannot speak about the direct responsibility of the genetic pathologies of Arc for the formation of one form or another of epilepsy in humans, but Arc can certainly be associated with epilepsy.

### 2.2. Expression of Arc protein in neurons activated by epileptic seizures

Arc depletion may affect memory loss in the post-ictal state. It is known that memory problems experienced by people with epilepsy are characterized by difficulty in retrieving episodes or events that happened before a seizure and even general semantic information (43). Genetic interference of activity-dependent Arc protein expression in the rat hippocampus impairs the maintenance of long-term potentiation and blocks the consolidation of long-term memory (33, 75). Can it be associated with the dysregulation of Arc protein expression during a seizure? It can be assumed that new methods of *in vivo* molecular imaging can help answer this question to some extent (76, 77).

Arc is an activity-dependent immediate early gene, its mRNA is translocated only to dendritic spines of active neurons where it is translated to Arc protein, which then multimerizes in capsids forming viral-like particles implanted with mRNA. Then in the form of viral-like particles, Arc-mRNA is loaded into extracellular vesicles and trafficked to postsynaptic boutons, participating in regulating dendritic spine morphology and the receptor content of glutamatergic synapses controlling synaptic plasticity changes and memory (20, 21, 31, 47, 78, 79). Notably Arc expression is augmented in synapses of recently activated neurons of the epileptic seizure focal zone but rapidly declines, probably due to dysregulation during aberrant neuronal overexcitation (80). For example, temporal lobe epilepsy originates from the mesolimbic network and provokes damage to the hippocampus including the dentate gyrus, which is very vulnerable to status epilepticus (81–83).

It is generally accepted that hippocampal sclerosis provokes the hippocampus to generate seizures resulting from the loss of interneurons and pyramids accompany the formation of recurrent synaptic circuits by dentate gyrus cells (DGC) (84, 85). Arc upregulation in DGC between seizures accompanies increased spine density which enhances excitatory input from the entorhinal cortex, which precedes the formation of recurrent mossy fiber synapses (80, 86). Conversely, hippocampal regions not affected by seizures preserve normal Arc expression (80).

Many authors studied the association between Arc expression and epileptogenesis. It is known that spine loss and other dendritic abnormalities occur in epilepsy which to some extent may be also associated with Arc (87). Of course, the Arc gene affects a whole galaxy of genes, and therefore the phenotypic consequences of its mutations are very diverse (21, 37, 88). Also, some genes and their products are necessary for Arc functioning and transport, and the disruption of these genes lead also to changes in neural net excitation (74).

### 2.3. Genetic studies of Arc expression during epileptogenesis

As part of the analysis of the role of Arc in epileptogenesis on clinical material, we analyzed the transcriptomic data from the National Center of Biotechnology Information (NCBI) public repository Gene Expression Omnibus (GEO) to evaluate the ARC expression in brain areas that are damaged in epilepsy (89). The RNA sequencing data were searched in the GEO Browser for the terms “epilepsy”, “epileptic”, and “seizure”. Afterward, we searched the GEO database for the terms “kainate”, “electroconvulsive”, and “pentylenetetrazol” to receive the data for the most common models of epilepsy. Each GEO dataset included in the analysis should meet the following criteria: (1) expression data in raw counts, fragments per kilobase per million mapped fragments (FPKM), or transcripts per million (TPM); (2) a clear explanation of sample origin (the datasets which comprise samples described like a “brain sample,” when the brain structure was not specified, were excluded); (3) at least three samples per study group; (4) the expression patterns were studied in brain structures, data for other tissues were excluded.

Four relevant datasets for the ARC expression pattern in human brain parts were mined (Table 1). In all datasets, only patients with temporal lobe epilepsy were included, and hippocampal structures were studied, except 17 neocortical temporal lobe samples in GSE134697. Further, no healthy or non-epileptic controls were included in the studies in the appropriate number (*n* ≥ 3) that did not allow for comparison of damaged tissues with normal tissues from healthy subjects.

**Table 1:** ARC expression in the temporal lobe samples of epileptic patients.

**Gene set ID**	**Title**	**Structure**	**Study group(s)**	**Expression levels (in CPM)**
GSE71058 (90)	Gene expression profiling in dentate granule cells from patients with mesial temporal lobe epilepsy with or without hippocampal sclerosis	*Gyrus dentatus*	12 patients with mesial temporal lobe epilepsy, including 5 samples from patients with hippocampal sclerosis and 7 without hippocampal sclerosis.	0–84.45
GSE94744 (91)	Microglia and the immune response to a human temporal lobe seizure	*CA1*	7 patients with mesial temporal lobe epilepsy and hippocampal sclerosis	1.01–28.62
		*CA3*		5.16–33.61
		*Gyrus dentatus*		3.20–18.02
		*Subiculum*		5.36–55.77
GSE127871	Altered expression of signaling pathways regulating neuronal excitability in hippocampal tissue of temporal lobe epilepsy patients with low and high seizure frequency	*Hippo- campus*	Investigation of alterations in hippocampal gene expression in temporal lobe epilepsy (< 4 seizure episodes per month vs. >4 seizures per month, 5 and 7 patients per group, respectively)	2.34–66.01
GSE134697 (92)	Hippocampal and neocortex transcriptome sequencing data from 17 mesial temporal lobe epilepsy patients and 2 neocortex samples from neurologically healthy controls	*Temporal neocortex*	17 mesial temporal lobe epilepsy patients	6.34–31.63
		*Hippocampus*		2.58–98.75

No differences in ARC expression levels in mesial temporal lobe epilepsy patients with or without concomitant hippocampal sclerosis were identified in GSE71058 by an edgeR likelihood ratio test (Padj > 0.05) (93). Likewise, there were no significant differences in ARC expression levels between patients with low and high seizure frequencies in GSE127871. All data were count per million (CPM) normalized by edgeR package CPM function and demonstrated the congruent ARC expression levels (93). Applying the Expression Atlas recommendations, the expression levels may be interpreted as low expression (i.e. CPM between 0.5 and 10) or medium (CPM between 10 and 1,000) in all studied structures (94).

In mice, transcriptome modifications were studied in the hippocampus, neocortical structures, and cerebellum in genetic models of epilepsy (Table 2). No differences in Arc expression levels between control and study groups were identified in any study when likelihood ratio test (for raw counts) or empirical Bayes statistics *eBayes* function in limma package (for log 2 FPKM-normalized data) were applied (99). The only exception is the dramatic loss of Arc in the hippocampal transcriptome of mice lacking miR-22 during the epileptogenesis after kainate injection compared to the identically treated wild-type animals (Padj < 0.05). miR-22 loss results in an exacerbated epilepsy phenotype in kainate-induced epilepsy and is associated with the reduction of an inflammatory response at the transcriptional level (96).

**Table 2:** Arc expression in genetic models of epilepsy in mice.

**Gene set ID**	**Title**	**Structure**	**Study group(s)**	Expression levels^*^	**Differential expression**	**Measure units**
GSE138370 (95)	Changes in calcium homeostasis and gene expression implicated in epilepsy in hippocampi of mice overexpressing ORAI1	*Hippocampus*	ORAI1 overexpressing group (*n =* 3) *vs*. WT^**^ (*n =* 3)	49.38–90.56	NS	CPM
GSE147466 (96)	Genetic deletion of microRNA-22 blunts the inflammatory transcriptional response to status epilepticus and exacerbates epilepsy in mice	*Hippocampus*	miR-22-/- (*n =* 4) *vs*. WT (*n =* 4); status epilepticus in both groups	44.47–75.63	Loss of Arc expression in miR-22-/– group (Pagj < 0.023)	FPKM
GSE151742 (97)	Expression of the neuronal tRNA n-Tr20 regulates synaptic transmission and seizure susceptibility	*Hippocampus*	B6N-n-Tr20-/- (*n =* 3) *vs*. B6N WT (*n =* 3)	66.48–161.59 (ribosomal), 66.58–128.52 (total)	NS	CPM
GSE169481 (98)	Deletion of a non-canonical regulatory sequence causes loss of Scn1a expression and epileptic phenotypes in mice	*Hippocampus*	heterozygous Scn1a KO (n=3) *vs*. WT (n = 4)	114.12–209.22	NS	CPM
GSE215425	WWOX P47T loss-of-function mutation induces epilepsy, progressive neuroinflammation, and cerebellar degeneration	*Prefrontal cortex*	WWOX P47T (loss-of-function mutation induces epilepsy, *n =* 5) *vs*. WT (n = 5)	43.70–698.65	NS	CPM
		*Parietal cortex*		34.47–496.00	NS	CPM
		*Hippocampus*		34.21–231.61	NS	CPM
		*Cerebellum*	WWOX P47T (*n =* 3) *vs*. WT (*n* = 4)	18.57–29.26	NS	CPM

^*^In control animals.

^**^NS, non-significant.

In contrast, the significant downregulation of Arc expression was identified in CA1 superficial layer in response to kainate treatment (Padj < 0.0019) in rats (Table 3). As well, Arc expression was downregulated in the dorsal subiculum (Padj < 0.0024) in response to the induction of acute seizures in rats by electric stimulation. No differences in Arc expression were identified between control and epileptic model rats when the whole hippocampus or *corpora quadrigemina* were studied. So, the demonstrated discrepancy between results revealed in mouse and rat models may be caused by expression examination in the whole hippocampus instead of the separated parts of this complex structure.

**Table 3:** Arc expression in non-genetic models of epilepsy in rat.

**Gene set ID**	**Title**	**Structure**	**Study group(s)**	Expression levels^*^	**Differential expression**	**Mea- sure units**
GSE137473 (100)	A systems approach delivers a functional microRNA catalog and expanded targets for seizure suppression in temporal lobe epilepsy	*hippocampus*	Perforant pathway stimulation (PPS) model (*n =* 3) vs. Ctrl (*n =* 3)	3.59–7.52	NS^*^	FPKM
GSE143555 (101)	RNA sequencing of laser-captured hippocampal deep and superficial CA1 subfields in epilepsy	*CA1*	kainic acid-induced status epilepticus (*n =* 3) and sham control (*n =* 3 group), deep and superficial CA1 subfields were studied separately	49,72–67,39 (superficial layer); 63.99–81.32 Ideep layer)	Downregulated in the superficial layer in kainite-treated animals (Padj < 0.0019)	CPM
GSE173885 (102)	Transcriptome of the audiogenic rat strain and identification of possible audiogenic epilepsy-associated genes	*corpora quadrigemina*	KM - audiogenic epilepsy rat strain (*n =* 3), Wistar rat (*n =* 4); outbred strain from KM rats (*n =* 4)	13.12–25.20	NS	CPM
GSE178409	Transcriptomic analysis of dorsal and ventral subiculum after the induction of acute seizures by the electric stimulation of the perforant pathway in rats	*subiculum*	Ventral and dorsal subiculum, after the induction of acute seizures by electric stimulation (*n =* 5) and in the control group (*n =* 5)	135.44–179.41 (dorsal); 30.91–63.16 (ventral)	Downregulated in dorsal subiculum in treated animals (Padj < 0.0024)	CPM
GSE193580	Hippocampus RNA-sequencing of Q808 against PTZ-induced seizure model	*hippocampus*	Rats were randomly divided into vehicle control group (*n* = 4), PTZ + vehicle group (*n* = 5), and PTZ + Q808 group (*n* = 5)	35.06–80	NS	FPKM

^*^In control animals.

^**^NS, non-significant.

Genetic studies of Arc expression during epileptogenesis in humans and animals are still rare to provide non-contradictory conclusions on Arc's role in seizure-related pathologies. According to available datasets for the Arc expression, only in mice and rats, but not in humans, the downregulation of Arc expression was revealed in response to seizures. Further accumulation of more detailed data on Arc expression in specific regions of the brain is likely to allow more specific conclusions to be drawn in the future.

## 3. Animal epilepsy model

### 3.1. Arc gene expression in rodent epilepsy models

Practically all rodent epilepsy seizure induction models affect Arc expression. In rodent models, Arc gene expression can be stimulated by practically any method which induces seizure activity in the brain, at least in the hippocampus and cortex, but Arc expression is time-dependent and the stimulation later turns to depression (80, 103, 104).

Initial hyperproduction of Arc can be seen in epilepsy models with kainate, D1-receptor agonists, 4-Aminopyridine (4AP), Bicuculline (Bic), and Forskolin, pilocarpine, pentylenetetrazole, kindling, activation of mGluR, electroconvulsive stimulation (Also Arc mRNA and proteins are rapidly induced in the striatum after acute cocaine administration (80, 105–111). Details on epileptic models inducing Arc expression are presented in Table 4.

**Table 4:** Animal epilepsy models and the role of Arc protein.

**Animal epilepsy models**	**Epilepsy symptoms and manifestations**	**Possible role of Arc in the control of epileptogenic activity**	**References**
**Non-genetic models**			
Mice' neuronal hippocampal and cortical cultures. Epileptogen's (4-Aminopyridine, bicuculline, forskolin) local administration	Synchronized network bursting in hippocampal cultures	Activity-induced Arc expression in neurons and astrocytes.	(106)
Temporal lobe epilepsy model (rats) pilocarpine-induced epilepsy	Status epilepticus	Optogenetics seizure control targeting intense ARC immunoreactive neurons Intense ARC immunoreactive neurons may have the potential to control epileptic seizures.	(112)
The mesial temporal lobe epilepsy model with the sclerotic hippocampus (mice) Optogenetically stimulated and kainic acid-induced epileptogenesis	Epileptiform events and status epilepticus in the hippocampus	The upregulation of Arc mRNA (1) is positively correlated with the epileptiform bursts, (2) increases during the increase of burst amplitudes and (3) increases with prolonged paroxysmal episodes. Arc is a possible mediator between synaptic plasticity and seizure activity.	(80)
Epilepsy after electroconvulsive shock treatment (rat) rat hippocampus and perirhinal cortex relationship between the current intensities that elicit seizures and the threshold for Arc mRNA transcription in the rat hippocampus and perirhinal cortex	Behavioral seizures with hind-limb extension and tonic–clonic motor responses	1. Intensive electrostimulation: the high proportion of Arc-positive neurons in the dentate gyrus, intermediate in the CA3 region of the hippocampus, lowest in the perirhinal cortex 2. Low-intensity electrostimulation: an opposite Arc expression profile (lowest in the dentate gyrus and highest in the perirhinal cortex)	(23)
Pentylenetetrazole-induced kindling in rats	The seizure intensity was classified according to the Racine scale	The most prominent increase in Arc expression during kindling was present in the entorhinal cortex, the dentate gyrus, and the basolateral nucleus of the amygdala	(107)
Intraperitoneal injection of pilocarpine to induce status epilepticus in rats	The seizure intensity was classified according to the Racine scale	Using Arc immunoreactivity as an indicator of granule cell activation, authors found that granule cells born after pilocarpine-induced SE did not express Arc more intensely than the surrounding granule cells and, in addition, transient seizure activity induced by pentylenetetrazol did not activate mature granule cells born after SE more intensely.	(110)
Electroconvulsive seizures in rats	Observation of generalized tonic/clonic seizure that lasted ~15 sec	Electroconvulsive seizures strongly induce prolonged Arc/Arg3.1 transcription in dentate granule cells. Assessment of Arc/Arg3.1 mRNA revealed that the induction of Arc/Arg3.1 transcription was blocked by NMDA receptor antagonists	(108)
Kainic acid-induced seizures in mice	Behavioral observation of the onset of seizure	Seizures elevated Arc/Arg3.1 protein in the granular cell layer and molecular layer of the dentate gyrus and in the pyramidal cells in CA1-3. The induction of a large number of activity-regulated genes, including Arc/Arg3.1, Arl5b, Gadd45b, Inhba, and Zwint, is indeed dependent on ERK phosphorylation.	(113)
**Genetic models**			
Angelman syndrome mouse model (mice). AS mice lack a functional copy of maternally inherited UBE3A but with a wild-type copy of the paternally inherited UBE3A allele.	Enhanced seizure-like response to an audiogenic stimulus	The reduction of the level of Arc expression has the potential to reverse the seizures associated with Angelman syndrome	(68)
Angelman syndrome UBE3A^m−/*p*+^ model in mice	Field potential recording in brain slices	Local circuits of UBE3A^m−/*p*+^ *in vitro* are hyperexcitable and display a unique epileptiform activity	(114)
Transgenic mice that express EGFP-Arc	A single generalized electroconvulsive tonic/clonic seizure that lasted approximately 15 s.	Arc mRNA degradation occurs via a mechanism with characteristics of nonsense-mediated mRNA decay (NMD). Rapid dendritic delivery of newly synthesized Arc mRNA after induction may depend in part on prior splicing of the 3′UTR.	(115)
EGFP-tagged Arc in the primary culture of hippocampal neurons	Switch from tetrodotoxin-induced inactivity to BDNF treatment	Activity-induced Arc/Arg3.1 accumulates at spines during synaptic inactivity. Synaptic Arc/Arg3.1 reduces surface AMPAR levels in individual spines.	(29)
Patients with idiopathic generalized epilepsy including childhood absence epilepsy and juvenile myoclonic epilepsy	Absence epilepsy	Authors suggest the presence of an idiopathic generalized epilepsy susceptibility allele in the ARC gene.	(116)
Mutant mice with the deletion of the Drd1a gene to prevent dopamine D1 receptor expression	Behavioral and EEG observation of seizures	Administration of D1-type receptor agonists promotes the expression of Arc/Arg3.1 in the hippocampal dentate gyrus. Deletion of Drd1a gene prevents the effect	(105)
Wistar Albino Glaxo from Rijswijk (Wag/Rij) rats	Absence epilepsy	Hippocampal mGlu5 receptor-dependent synaptic plasticity is associated with the pathological phenotype of WAG/Rij rats. Arc is involved in mGluR-induced long-term synaptic depression (mGluR-LTD)	(117–119)
Arc–/– and Arc+/–mice	Electrical stimulation of dopamine neurons in the midbrain ventral tegmental area and Ca-imaging	Genetic disruption of Arc leads to concomitant hypoactive mesocortical and hyperactive mesostriatal dopamine pathways.	(120)
Arc^−/−^ Mice Have Decreased Spine Density and Increased Spine Width. Kainite model	*Arc^−/−^* mice are more susceptible to seizures in response to systemic challenges with pentylenetetrazol (PTZ).	Arc specifically reduces surface GluR1 internalization at thin spines, and Arc mutants that fail to facilitate AMPAR endocytosis do not increase the proportion of thin spines. Loss of Arc *in vivo* leads to a significant decrease in the proportion of thin spines and an epileptic-like network hyperexcitability.	(21)

Arc expression demonstrates complex behavior in seizure generation. The threshold of stimulation intensity (depolarization threshold) required for induction of Arc expression varies between brain regions (23). Particularly in the case of most intensive electroconvulsive stimulation, the proportion of Arc-positive neurons following seizures was highest in the dentate gyrus, intermediate in the CA3 region of the hippocampus, and lowest in the perirhinal cortex (23). In contrast, low-intensity seizure-inducing electrostimulation caused an opposite Arc expression profile (lowest in the dentate gyrus and highest in the perirhinal cortex), which indicates for Arc expression may be serving as a transcriptional threshold mechanism in CNS (23). Accordingly, Arc mRNA upregulation is positively correlated to seizure burst ratio, burst amplitudes, and length of paroxysmal episodes (80).

### 3.2. Rodent models with Arc knock-out

Peebles et al. studied spine morphology and the general stability of the glutamatergic neuronal network and found both dependent on Arc expression, employing a mouse model (21). They have confirmed that Arc expression leads to an increase in spine density, but generally decreases synaptic efficacy by reducing surface GluR1. Authors have shown that regulated synaptic strength in neuronal networks determined by Arc is very important for network stability. Studying kainite-elicited seizure activity in WT and Arc–/– mice, authors have shown that Arc–/– mice are more susceptible to kainite-elicited seizures and neuronal changes associated with epilepsy. Also, Arc–/– mice had aberrant spontaneous cortical network discharge activity, highly associated with epilepsy (21). On the other side, prenatal or perinatal deletion of *Arc/Arg3.1* alters cortical network activity without excessive disruption of the balance of excitation and inhibition in the brain (88). Furthermore, Arc knockouts elevate AMPA receptor level expression in some brain regions including the nucleus accumbens which reduces the symptoms of epilepsy-associated pathologies (109). Pilocarpine-induced temporal lobe epilepsy causes a time-dependent decrease in Arc expression in hippocampal tissue (121).

Epilepsy is prevalent and often medically intractable in Angelman syndrome (AS). There are different models of AS. AS mouse models associated with UBE3A gene-deficient function (UBE3Am-/p+) shows reduced excitatory neurotransmission but a lower seizure threshold. Genetically decreased Arc expression additionally reduces abnormal EEGs and seizures in mice with Angelman syndrome associated with UBE3A gene-deficient function, because both Arc and UBE3A regulate surface expression of AMPA receptors. In another AS model, the so-called fragile X syndrome (FXS) mouse model, on the contrary, increased Arc is responsible for seizure phenotype (68, 114).

Comorbidity of schizophrenia and epilepsy are relatively common in clinical practice and animal models. Two different models of schizophrenia with seizures were developed, conditional KO (late-cKO) mice, in which Arc/Arg3.1 was deleted during late postnatal development, to investigate the causal relationship between *Arc/Arg3.1* deletion and schizophrenia-linked neurophysiological and behavioral phenotypes. Nevertheless, in an animal study genetic deletion of Arc/Arg3.1 *per se* did not cause schizophrenia-like behavior, and a significantly higher dosage of kainic acid was required to elicit epileptic seizures in the KO mice (88). This completely contradicts the results obtained in another model of Arc/Arg3.1 knockout mice by Managò et al. showing genetic mutation disrupting Arc produced a hyperactive phenotype and amphetamine supersensitivity consistent with rodents' correlates of schizophrenia-like symptoms (120). The authors also describe important differences in the dopaminergic system in KO and wild-type mice. Most likely, this inconsistency can be explained by the different strategies used to create the two knockout mouse models.

## 4. Discussion and conclusion

**Figure 2:**
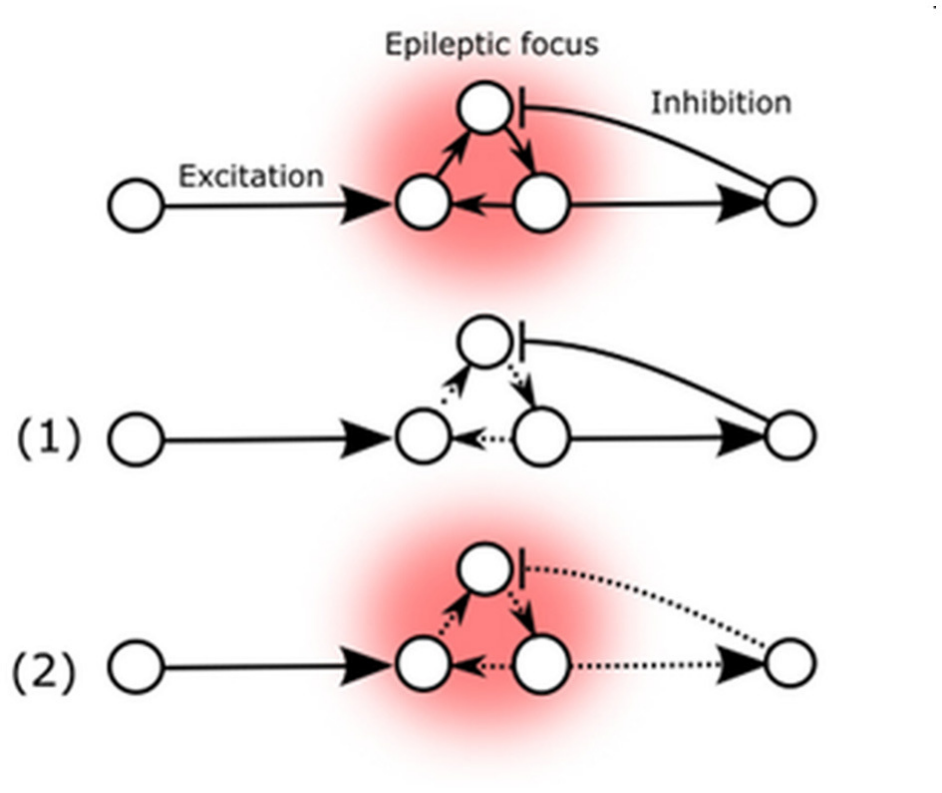
Arc mediated changes of seizure susceptibility. (1) Elevated Arc expression in epileptic focus weakens external AMPA-mediated excitatory influence. (2) Arc expression also decreases excitatory feedback to inhibitory neurons.

The literature sources cited unanimously emphasize the paramount significance of Arc protein in regulating synaptic strength [reviewed by Zhang and Bramham (122)], which in turn determines the stability of the neuronal network, thus affecting epileptic seizure susceptibility (Figure 2) (21, 122). While the established mechanism of Arc-mediated influence relies on presynaptic NMDA-receptor mediated ionic currents in the glutamatergic pathway, it is also the primary mechanism that exerts direct cross-synaptic influence over postsynaptic spines morphology and their AMPARs (21). These spines are typically found at the postsynaptic site of most excitatory synapses in the mammalian brain and may be present on excitatory as well as inhibitory neurons (123). This implies that changes in Arc-protein-mediated activity can potentially affect the excitatory-inhibitory balance of the neuronal network, either reducing seizure threshold and promoting stability or inducing lability (88, 114).

These two possibilities are represented by the potential Arc-mediated changes (Figure 2). The primary known effect of the Arc protein is to decrease the strength of excitatory synapses, which could potentially reduce the destabilizing flow of excitatory signals to the existing epileptic focus or decrease auto-excitation by reducing internal excitatory connections (1). Conversely, Arc-mediated changes could also decrease excitatory feedback to inhibitory neurons, thereby reducing their inhibitory influence on the existing epileptic focus (2).

Arc knockouts will remove such effects: the literature reviewed and knockout experiments consistently show this two-sided effect on seizure susceptibility, if some existent epileptiform activity is present. While Arc definitely is activated in neurons participating in epileptic discharges, there is no evidence of the direct induction of epilepsy by Arc-protein mediated mechanisms.

These allow us to conclude that Arc activity-regulated expression directly participates in seizure susceptibility. Also, epileptic activation of Arc-mediated changes during the seizure may affect memory consolidation and many other important neuronal functions in epilepsy.

## Author contributions

Conceptualization: DS, MI, AV, ANV, and VT. Writing: MI, DS, AI, AV, ANV, and VT. Review and editing and figures preparation: DS, AI, and MI. Editing: JA, AI, ES, LR, and PS. Supervision and project administration: MI, VT, and AV. Funding acquisition: MI, AV, and ANV. All authors have read and agreed to the published version of the manuscript.
